# Contribution of Fruits and Vegetables to the Household Food Security Situation of Rural Households in Limpopo

**DOI:** 10.3390/nu15112539

**Published:** 2023-05-29

**Authors:** Zoleka Sithole, Muthulisi Siwela, Temitope Oluwaseun Ojo, Simphiwe Innocentia Hlatshwayo, Richard Jack Kajombo, Mjabuliseni Simon Cloapas Ngidi

**Affiliations:** 1 Discipline of Dietetics and Human Nutrition, School of Agricultural, Earth and Environmental Sciences, College of Agriculture, Engineering and Science, University of KwaZulu-Natal, Private Bag X01, Scottsville, Pietermaritzburg 3201, South Africa; 2 Centre for Transformative Agricultural and Food Systems, School of Agricultural, Earth and Environmental Sciences, College of Agriculture, Engineering and Science, University of KwaZulu-Natal, Private Bag X01, Scottsville, Pietermaritzburg 3201, South Africa; 3 Department of Agricultural Economics, Obafemi Awolowo University, Ile-Ife 220101, Nigeria; 4 Disaster Management Training and Education Centre for Africa, University of the Free State, Bloemfontein 9301, South Africa; 5 Economic Planning and Development, Capital Hill, Economic Planning Building, Lilongwe P.O. Box 30136, Malawi; 6 Department of Agricultural Extension and Rural Resource Management, School of Agricultural, Earth and Environmental Sciences, College of Agriculture, Engineering and Science, University of KwaZulu-Natal, Private Bag X01, Scottsville, Pietermaritzburg 3201, South Africa

**Keywords:** food security, fruits and vegetables, household food insecurity access scale (HFIAS), Poisson regression model, endogenous treatment model

## Abstract

Food insecurity continues to be a burden for many South Africans. The production and consumption of fruits and vegetables have a potential role in improving household food security and are considered one of the critical pathways for reducing food insecurity and malnutrition levels in the country. This paper set out to determine the effect of fruits and vegetables on the food security status of rural households in the Limpopo province. Data (secondary) for this study were collected from 2043 respondents who were selected through stratified random selection based on the population size of the district municipalities in Limpopo. This study used a quantitative research approach, and data were analyzed using a descriptive analysis, the household food insecurity access scale (HFIAS), and a Poisson regression model with an endogenous treatment model. The findings revealed that gender and involvement in agricultural production had a positive significant relationship with the consumption of fruits and vegetables, while disability grants had a negative impact. Age, household size, and receiving a disability grant had a positive significant impact on determining the household food insecurity status; however, gender had a negative significant relationship. This study concluded that the consumption of fruits and vegetables considerably influenced the food security status of the household. There is a need for government officials and local leaders to provide food security interventions that prioritize women and elders. These may include promoting household production and consumption of diversified fruits and vegetables.

## 1. Introduction

Food security is and has been one of the strategic imperatives for the South African government and policy makers. This is expressed in many governments’ policy documents, including the constitution and the national development plan. The right to have access to sufficient food for all citizens is enshrined in the constitution of the country. While South Africa is nationally food secure, several households face food security challenges [1,2]. The state of food and nutrition vulnerability in South Africa has been exacerbated by economic hardship due to high rates of unemployment and the outbreak of COVID-19, with associated control measures implemented by the government to contain its spread. GHS 2020 revealed that between 2019 and 2020, the proportion of households and individuals that were vulnerable to hunger increased from 10.3% to 10.8% and 11.1% to 11.6%, respectively [3]. In the same period, the number of households and individuals who experienced difficulties with food access also increased by 2.8% and 2.5%, respectively.

Agriculture plays an important role in the process of economic development and can significantly contribute to household food security. Although several policy decisions and programs have been implemented to address food and nutrition insecurity over time, programs promoting the production and consumption of fruits and vegetables still need special attention, as the literature has shown that many South African do not produce and consume vegetables [1,4]. In 2020, only 17.5% of South African households were reportedly involved in agriculture. Of the nine provinces of South Africa, Limpopo had the highest proportion of households engaged in agriculture, with 37.5% of the households involved in agriculture [3]. Generally, fruits and vegetables are not produced by higher numbers of households, let alone consumed by them. However, agriculture, including fruits and vegetables, may contribute positively and directly to improving the food security status [5,6].

Nutrition-sensitive agriculture or the production of foods with high nutrient densities (such as dairy, fish, fruit, meat, and vegetables), is recognized as a pathway to improve food and nutrition security [6]. As noted by Vinceti et al. [7], fruits and vegetables could address the need for nutritious and adequate food in the context of many challenges facing South Africa and Africa in general, including reducing poverty and hunger, improving environmental health, enhancing human well-being and health, and strengthening local food networks, sustainable livelihoods, and cultural heritage.

Fruit and vegetable production contributes to household food security by providing direct access to food that can be harvested, cooked, and served to family members, usually daily [8]. Even the poorest, most landless, or near-landed people are accustomed to farming in small plots of land, uninhabited areas, sidewalks or the edges of fields, or in containers. Farming can be accomplished without economic resources, using locally available planting materials, green manure, “living” fences, and traditional pest control methods. Farming provides a variety of new foods that improve the quantity and quality of the nutrients that are available to a family. Increasing fruit and vegetable production is an obvious first step [5].

Growing population figures and rising wages, especially in urban areas, have created an increase in market demand as consumers seek to diversify their food intake. Increasing vegetable production to meet this need creates significant economic opportunities, especially for smallholder farmers. Market-focused vegetable farming not only creates income for smallholder farmers but it also helps to build their resilience to external risks. The diversity of vegetable crops, short growing cycles, and efficient use of irrigation can reduce farmers’ risk of climate change. To grow the economy, farmers can choose to incorporate vegetables into existing cropping systems or specialize in vegetable production [5,9]. Furthermore, providing the important micronutrients needed for a healthy diet, improving livelihood, and decreasing health issues. Considering this background, this study investigates the contribution of fruits and vegetables to household food security.

## 2. Methodology

### 2.1. Description of Study Areas

**Figure 1:**
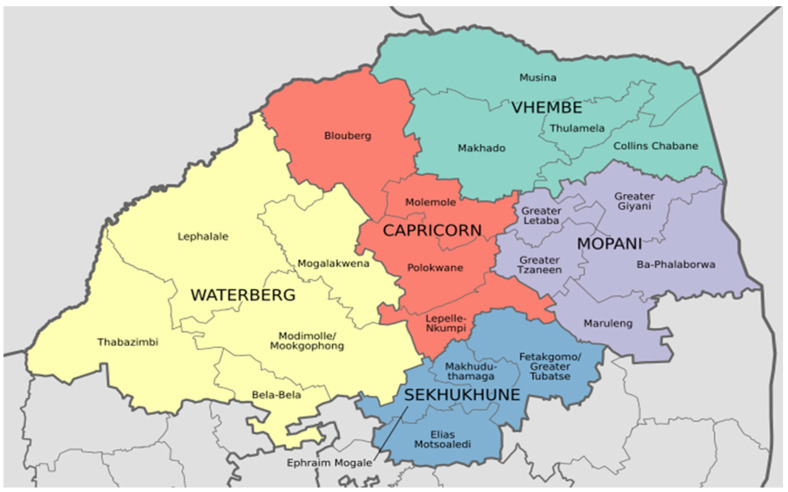
Limpopo province districts and their municipalities covered in the study.

A cross-sectional descriptive study was conducted in the northern part of South Africa, in the Limpopo province, which covers about 125,754 km^2^ of the country’s total area. Its population is about 5.8 million, with 1.6 million households and five districts named Mopani, Vhembe, Capricorn, Waterberg, and Sekhukhune [3,10]. Figure 1 shows Limpopo province districts and their municipalities. Lehohla [11] reported that in Limpopo, 68% of the land is capitalized for the agricultural sector (emerging crop, subsistence, commercialized, and livestock farming). Limpopo is the most food-secure province in Mzansi, with households highly involved in agriculture for survival [3]. Although the province is known for high weather temperatures ranging from 45 °C to 50 °C during the summer, resulting in drought, agriculture production is still functional [12]. The study included farmers and rural and urban community dwellers.

### 2.2. Data Collection Method

The study used secondary data, which were collected using stratified random sampling based on district population size in 2020, from a total of 2043 respondents to gain insight into the food and nutrition security of Limpopo rural households. To gather the information, structured questionnaires were used; information included demographics, subsistence (food availability, which included food production, consumption, and sold by household), the household food insecurity access scale (HFIAS), shocks and social networks, and food insecurity experience due to the impact of COVID-19. The data used in this paper were extracted from this data to obtain a comprehensive understanding of the effect of fruits and vegetables on food security in rural households. Table 1 shows the list of available fruits and vegetables to smallholder farmers.

This study used a quantitative research approach, and the cluster sampling technique was used to select household heads from different municipalities and districts. The cluster sampling method is one of the random sampling methods that has a time- and cost-effective sample size. It is easily accessible and increases efficiency and validity, which was advantageous for this study as the population was widely spread among Limpopo provinces. Furthermore, it decreases variation [13].

### 2.3. Data Analysis

The study used Statistical Package for the Social Sciences (SPSS) Version 20 to analyze and compare data. Descriptive statistics, which include the means, standard deviations, frequencies, and percentages, were computed where applicable. The version uses variables of the respondents to analyze demographics and measure the household food insecurity status in Limpopo. The household food insecurity status was assessed using the food security indicator: the household food insecurity access scale (HFIAS).

The household food insecurity access scale (HFIAS) is a continuous indicator for measuring household food insecurity status. This scale was initially handed down by USAID’s Food and Nutrition Technical Assistance (FANTA) project [14,15]. The household food insecurity access scale (HFIAS) comprises nine structured questions representing the universal domain of exposure and vulnerability to food (access) insecurity in the past four weeks. Participants are limited to 3 possible responses: never, sometimes, and often (1, 2, and 3), respectively. The questions come up from a range of 0 to 27. As the scoring increases, the likelihood of a household experiencing food insecurity increases, and the lower the scoring, the lower the exposure to food insecurity, and vice versa. The food security status depends on the frequency of occurrence. The HFIAS is also used to calculate the household prevalence of HFIAS, which is categorized into 4 groups, namely, food security, mild food insecurity, moderate food insecurity, and severe food insecurity [14,15,16].

The study’s objective is to assess the contribution of fruit and vegetables to household food security. Therefore, it is assumed that rural households that produce and consume fruit and vegetables have the means to access financial resources that will help them obtain other nutritious foods to meet their daily food requirements. An investigation of the impact of treatment selection (fruit and vegetables) on the outcome variable, in the jargon of impact assessment. The HFIAS is the outcome variable, defined as a continuous measure of the household’s degree of food insecurity (access) in the past four weeks (30 days). Households that consumed and produced fruit and vegetables receive a score of 1; otherwise, they receive a score of 0.

Subject characteristics commonly influence treatment selection in an observational study like this. Farmers usually make voluntary decisions to produce and consume fruit and vegetables based on their socioeconomic factors and production capacity, which results in self-selection bias. In this scenario, farmers’ production and consumption of fruit and vegetables cannot be assigned at random. When households are not handled randomly, their decisions to produce and consume fruit and vegetables can be influenced by observed and unobserved factors that correspond with the outcome variables. Another critical economic obstacle in impact evaluation is the issue of missing counterfactual data. Data are missing because outcomes can only be observed in one state, and counterfactuals for each group cannot be observed [17].

This study employed the Poisson regression model, which was also employed by Danso-Abbeam, Ojo [18]. The model estimates the causal effect of fruit and vegetables on the household food security by means of the count outcome with a Poisson distribution of the error term. The primary goal of this study is to determine the average treatment effect on the treated (ATT). Takahashi and Barrett [19] define ATT as the average difference in potential outcomes of smallholder farmers who have produced or do not produce fruit and vegetables. The ATT can be represented as follows, according to Imbens [20] and Adolwa et al. [21].

(1) $$ATT=E\left(Y_{1j}-Y_{0j}/\;T_{j}=1\right)=E\left(Y_{1j}/T_{j}=1\right)-E\left(Y_{0j}/T_{j}=1\right)$$

where E{.} denotes the expectation operator; $Y_{1j}$ is the potential outcome for households who produce and consume fruit and vegetables; and $Y_{0j}$ is the potential outcome of households who do not produce and consume fruit and vegetables. $T_{j}$ represents the treatment indicator, which takes the value 1 if smallholder farmers consumed fruit and vegetables, and 0 otherwise. Unobserved counterfactual events pose a significant barrier to predicting the ATT. As a result, observing the prospective consequences of farmers who produced or consumed fruit and vegetables is nearly impossible. Replacing this unobserved counterfactual with the possible results of smallholder farmers who have not produced or consumed any fruit or vegetables is similarly impractical because it is likely to result in biased estimations primary model; endogenous Poisson treatment effect is used to address this problem.

#### Endogenous Treatment Effect Model for a Count Outcome—Poisson

As previously stated, the study intends to see if fruit and vegetables affect household food security status. Because the production or consumption of fruit and vegetables by smallholder farmers is not exogenous, it is regarded as an endogenous binary-treatment variable $T_{j}$. $T_{j}$ is endogenous if the treatment assignment is not random, but some unobservable covariates (variables) are affecting $T_{j}$ that also influence the outcome variable. Since the HFIAS (outcome variable) is a count event that takes values, $Y_{j}=0,1,2,.....Y_{n}$ and smallholder farmers choose to adopt one or none, a second dummy $S_{j}$ was developed to represent a sample selection rule. That is, smallholder farmers may not be able to consume or produce fruit and vegetables. In this case, $S_{j}$ is missing for a proportion of the sample and the selection rule is defined as $S_{j}=1$ when $Y_{j}$ is observed and $S_{j}=0$ when $Y_{j}$ is missing. Endogeneity and sample selection were solved using the count data model with endogenous treatment [22].

The Poisson endogenous treatment effect model regards the case where selection dummy $S_{j}$ is assigned the value 0 when smallholder farmers are not food secure ($Y_{j}$ is missing), and 1 when smallholder farmers are food secure ($Y_{j}$ is observed). Selection dummies and endogenous treatment can be produced using continuous latent variables such as the following:

(2) $$T_{j}^{*}=Z_{i}^{'}+\mu_{j}$$

(3) $$S_{j}^{*}=X_{j}^{'}\beta +\delta T_{j}\;+\varepsilon_{j}$$

With $T_{j}=1(T_{j}^{*}>0),S_{j}=1(S_{j}^{*}>0)$, the outcome model that follows a Poisson distribution can be specified as follows:

(4) Yj={{0μYjexp(−μ)}/Yj!  if S=1if S=0}

Thus,

(5) $$E(Y_{j}/X_{j},T_{j},\varepsilon_{j})=\;\exp(X_{j}\beta +\delta T_{j}+\varepsilon_{j})$$

$X_{j}$ indicates the covariate vector used to model the count outcome; $Z_{j}$ is the covariate for binary treatment; and $\varepsilon_{j}$ and $\mu_{j}$ are the error terms for the outcome and treatment, accordingly. The two error terms have a mean of zero and are bivariate normal. Since the covariates $X_{j}$ and $Z_{j}$ are exogenous, they are unrelated to the error terms. Conditional on $\varepsilon_{j}$, $\mu_{j}$ is normal with mean $\varepsilon_{j\rho /\sigma }$ and variance $\left(1-\rho^{2}\right)$. The endogenous treatment Poisson regression model is nested in a possible outcome model to estimate the ATE and ATT. The prospective outcome model describes what each farm household might receive at each treatment level. Table 2 summarizes the variable names, definitions and expected signs.

## 3. Results

### 3.1. Demographic Characteristics

Table 3 represents an overview of the demographic characteristics of the study participants. Out of the 2043 participants, 98.9% (n = 2020) of the household were African, 0.9% (n = 18) were white, and 0.2% (n = 4) were other groups colored. The study was dominated by female household heads (53.8%), and there were only 46.2% males. The minimum household head age was 18, and the maximum was 103, with a mean of 53.90 years. The minimum and maximum household sizes were 1 and 24, respectively, with a mean of 4.59. About 92.9% of household members lived in formal dwellings.

The education level of household heads ranges from grade 0 to doctoral degree; however, the results show that most households were headed by an individual that acquired grade 8/std 6 to grade 12/std 10 (42.2%). Only 0.2% (n = 4) had a doctoral degree, and 18.2% (n = 310) reported that they never went to school. About 70.7% (n = 1055) were unemployed, and 15.4% (n = 230) were employed full time. The majority of participants were working in farms (6%), education (5.4%), manufacturing (5.2%), and other workplaces, respectively. A greater percentage (38.9%) of households had an income that was between R1501 and R300. Moreover, 38.0% of household heads were legally married, and 27.9% were single (never been married). Surprisingly, 88.9% of households owned land, and 82.5% of the land was used to produce food and other agricultural products.

### 3.2. Availability of Fruits and Vegetables in Limpopo

Table 4 shows fruits and vegetables grown and consumed by rural households in Limpopo provinces. The results revealed that 82.5% of the land was used to produce food and agricultural activities. The results also showed that 96.8% of households consumed fruits and vegetables, while only 24.6% were involved in fruit and vegetable production. About 96.8% of rural households consumed vegetables, while only 21% were involved in their production. Regarding fruit production and consumption, 32.6% of households consumed them, while 10.5% produced them.

### 3.3. The Prevalence of Food Insecurity among Sampled Limpopo Households

**Figure 2:**
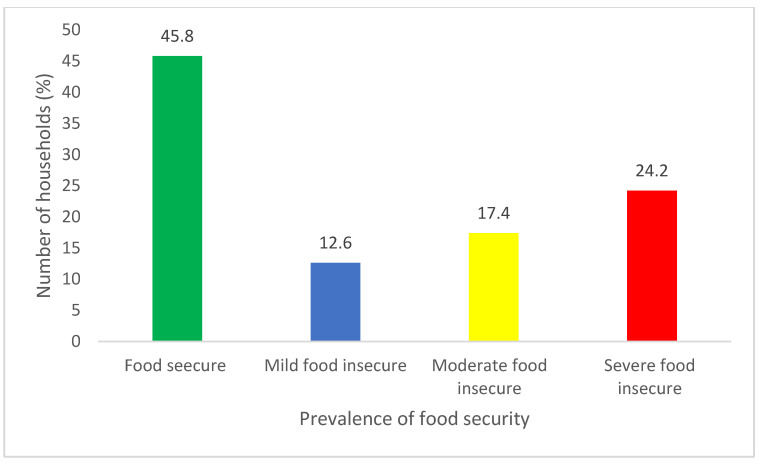
The food insecurity situation of rural households in Limpopo province.

The prevalence of household food insecurity was determined using HFIAS categories presented in Figure 2. The analysis for food (access) insecurity status indicated that 45.8% (n = 616) of households were food secure, followed by 24.4% who were severely food insecure, and 17.4% were moderately food insecure. Only 12.6% of the households were mildly food insecure. These results show that some households still experience difficulties in accessing healthy and nutritious food.

### 3.4. Access to Food Insecurity Determined by the Household Food Insecurity Access Scale (HFIAS) with Endogenous Poisson Regression Model

The Wald Chi^2^ (24.92, *p* > 0.000) shows that the model is statistically significant at 1%, indicating a good fit. The rho (ρ) was statistically significant at 1% (0.641, *p* > 0.002). The significance of the rho *(ρ*) indicates that there were unobserved characteristics of rural households that influenced their decision to produce and consume fruits and vegetables and also affected their food security. The Poisson endogenous treatment effect model should be used to solve the endogeneity issue. The results showed that age, gender, household size, social grant, involvement in agricultural production, and social relief for disability were all statistically significant, as shown in Table 5.

The results indicated that age did not influence the consumption of fruits and vegetables; however, it had a positive impact and was statistically significant for household food insecurity. Gender had a positive impact and was statistically significant for the consumption of fruits and vegetables, while it had a negative and significant impact on household food insecurity. Household size positively impacted household food insecurity, and it was statistically significant at the level of 5%. Social relief for disabilities did not significantly impact the consumption of fruits and vegetables; however, it showed a positive and significant impact on household food insecurity. The results further revealed that the grant recipient’s household head had a negative impact and was statistically significant (*p* > 0.10) for the consumption of fruits and vegetables. When the household head received the grant, more fruits and vegetables were consumed, yet it did not significantly influence the household food insecurity status. Involvement in agricultural production had a positive impact and was statistically significant for the consumption of fruits and vegetables, while it did not have any significant impact on household food insecurity.

#### Treatment Effects on Production and Consumption of Fruits and Vegetables

A simple, considerable difference in the production and consumption of fruits and vegetables in effect assessment is misleading, as it involves bias and it fails to consider the potential heterogeneity in the characteristics of rural households. Therefore, this study turned to the results of the effects of fruits and vegetables on household food security in terms of HFIAS using ATT and ATE, where the Poisson regression with endogenous treatment effects was used. The ATE and ATT were assessed after fitting the Poisson regression with endogenous treatment effects. As shown in Table 6, the estimated potential outcome mean (ATE) of fruits and vegetables on household food security was about 5.103 and was statistically significant at 1%. The ATE estimate indicated that the average rural households who consumed and produced fruits and vegetables had improved food security. Similarly, the conditional treatment effect, which measures the ATT of the contribution of fruits and vegetables to food security, was about 6.371 and statistically significant at 1%. Therefore, rural households who consumed and produced fruits and vegetables had an average of about 6.371 more improved food security than those who did not consume and produced them.

## 4. Discussion

The study’s objective was to investigate the contribution of fruit and vegetable consumption to food security in the Limpopo province of South Africa. The study aimed to understand the relationship between the consumption of fruits and vegetables and the factors that influence the consumption of fruits and vegetables and the food security status of households. The study revealed that very few households consumed and produced fruits and vegetables; only (n = 708) consumed fruits and vegetables and (n = 485) produced fruits and vegetables. These results were significantly low and surprising, as it is known that fruits and vegetables are crucial for a diversified, balanced, and healthy diet [23,24,25]. In addition, the study found that the majority of Limpopo households were food insecure (54.2%). This is due to many determinant factors that influence food access and consumption. These results align with several studies [2,18,26] conducted in Limpopo regarding the food security status of households; these studies revealed that most households were food insecure. Although Limpopo is known to be a food-secure province in South Africa [3], many rural households still suffer from food insecurity.

The findings revealed that age had a negative impact on the consumption of fruits and vegetables, yet it was not significant. However, it had a positively significant relationship with the household food insecurity status., i.e., as the age of the household head increases, the consumption of fruits and vegetables decreased and the probability of being food insecure increased. This may suggest that older individuals were not consuming a nutritionally balanced diet. Being old decreases their chances of accessing food and consuming fruits and vegetables. These affect their daily nutrition requirements, hence compromising individual health. This can be justified by other studies that revealed that as one’s age increases, most members become less active, and there is poor involvement in food production and preparation [27,28,29]. Even though most older household heads are decision-makers of the household and they manage resources, Smith et al. [28] noted that older farmers are mostly food insecure due to their increased dependence. In addition, Hall et al. [30], Oliveira et al. [31], and Xaba et al. [32] found that increasing age was associated with low fruit and vegetable consumption. These studies found that adults had poor consumption of fruits and vegetables compared to younger household members. Unlike the study conducted by Awobusuyi et al. [33] in Nigeria, the authors found contradicting results that concluded that older household heads were more food secure than younger household heads, which justified as being older as being associated with more knowledge and farming experience.

The household head’s gender showed a significant relationship between the consumption of fruits and vegetables and the food insecurity status. Gender positively impacted the consumption of fruits and vegetables, i.e., being male was associated with increased consumption of fruits and vegetables compared to females. This may be due to the reports that females in rural households generally have more responsibilities, which include taking care of the family, household chores, meal preparations, and food distribution. They have less time to take care of themselves and eat healthy foods. Males are more prioritized as heads of the family; as a result, females have less access to food as they first distribute to the family [30,34]. These results are in contrast with studies conducted by Xaba et al. [32], Dehghan et al. [34], Simunaniemi et al. [35], and Tamers et al. [36], who found that female-headed households had better fruit and vegetable consumption than male-headed households. Moreover, in this study, gender negatively impacted household food insecurity, implying that female-headed households were less food insecure. This is because women are regarded as pillars of the household; they are involved in agricultural production, care, and preparing and planning meals for their families [29,37,38]. These findings contradict the findings obtained in the study conducted in Niger, Brazil, which showed that male-headed households were more food secure than female-headed households [39,40]

The household size (household members) influences the household structure, function, food demand, availability, and consumption. This study revealed that the household size positively influenced the consumption of fruits and vegetables but was insignificant. Nevertheless, household size had a positive significant impact on the household food insecurity status., i.e., as the members of a household increases, the risk of being food insecure increases, hence the decreasing consumption of fruits and vegetables. To feed a household with a large number of family members, more resources and money are needed to buy healthy foods [38,41]. These findings align with the study findings by Rubhara [42], who found a positive relationship between the household size and household food insecurity. The study concluded that an increase in household members is directly related to an increase in demand and supply, respectively; hence, the bigger the household, the more food insecure they are, and the fewer household members, the less food demand, and vice versa. On the contrary, the study conducted by Zondi et al. [29] found that bigger household size increased food security status. Families with many household members can divide duties among each other in production or agricultural activities. In return, they can increase the food produced for consumption and surplus, which can generate income; hence, households consume a more diversified diet and increase their food availability [43].

The results of this study also revealed that receiving grants had a negatively significant relationship with the consumption of fruits and vegetables, but it was not significant for the household food insecurity status. Additionally, receiving a social relief grant for disability had a positively significant relationship with the household food insecurity status, i.e., receiving grant decreased the level of consumption of fruits and vegetables while receiving social relief for disability increased the likelihood of being food insecure. Initially, the purpose of giving grants in SA was to ease household financial constraints and access basic needs to better their livelihood and living standards. However, due to high levels of unemployment, grants are used as the main financial support or income for households and their extended family and they cover household expenses. As food prices rise drastically, having good quality, quantity, and diversified food becomes a challenge, therefore increasing the level of food insecurity among households [24]. These findings are in line with other studies by Okop et al. [24], Zondi et al. [29], Aliber [44], and Patenaude et al. [45], which all found that receiving the grant was associated with increased food insecurity. These studies revealed that many households in SA depend on grants or disability grants to maintain and sustain their household.

As expected, involvement in agricultural production had a positively significant relationship with the household consumption of fruits and vegetables., i.e., involvement in agricultural production increased the household consumption of fruits and vegetables. Involvement in agricultural production (includes the production of fruits and vegetables) increases food availability, improves dietary diversity, and increases income opportunities. This is consistent with findings from previous studies that have reported a good correlation between involvement in agricultural production and diet quality [6,46,47,48].

## 5. Conclusions and Recommendations

The role of fruits and vegetables in improving livelihoods and food security is underrated, although food insecurity continues to be a cause for concern for the government and policy makers in South Africa. Increasing the focus and promotion of the production and consumption of fruits and vegetables is a possible pathway for reducing the country’s high food insecurity levels. Therefore, this paper studied the contribution of fruit and vegetable consumption and the food security status of Limpopo province households. While households consumed and produced fruits and vegetables, more households were involved in the consumption of fruits and vegetables than production.

The consumption of fruits and vegetables considerably influenced the food security status of the household. Additionally, age, household size, and receiving social relief for disability grants had a positively significant impact on determining the household food insecurity status; however, gender had a negatively significant relationship. Numerous factors such as age, gender, household size, social grants, and involvement in agricultural production affected the consumption of fruits and vegetables. Gender and involvement in agricultural production had a positively significant relationship with the consumption of fruits and vegetables, but being a recipient of a grant had a negative impact. There is a need for government officials and local leaders to provide food security interventions that prioritize women and elders. These may include promoting household production and consumption of diversified fruits and vegetables.

### Study Limitations

This paper focuses on the overall provincial food security situation. Further studies could be conducted to show the disaggregated district and municipal results. This will enable policy makers to enact district- and municipality-level-specific interventions.

## Figures and Tables

**Table 1:** Types of available fruits and vegetables in Limpopo province.

Fruits	Vegetables
Pear	Spinach
Apricot	Imifino
Mango	Morogo
Pawpaw	Beetroot
Sweet melon	Brinjals
Yellow flesh peach	Broccoli
Yellow flesh plums	Brussels
100% fruit juice	Sprouts
Apple	Cabbage
Banana	Cauliflower
Fig	Gem squash
Avocado	Green beans
Granadilla	Onion
Grapes	Peas
Guava	Tomato
Lemon	Turnip
Maroela	Thepe
Melon	
Orange	
Guava	
Pineapple	
Strawberries	
Plum	

**Table 2:** Definition of variables and prior expectation.

Variable	Definition	Expected Sign
Age	Continuous: total number of years.	±
Education of household	Categorical: grade 0/R to doctoral degree.	+
Employment status of household	Categorical: 1 = employed full time; 2 = employed part time; 3 = self-employed; 4 = not employed; 5 = studying full time.	+
Gender	Dummy: 1 for male and 2 for female.	±
Household size	Continuous: number of members of the household.	±
Did work for wages/salary	Dummy: 1 = yes; 2 = no.	+
Household grant recipient	Dummy: 1 = yes; 2 = no.	±
Receive any social relief of disability	Dummy: 1 = yes; 2 = no.	±
Access to land	Dummy: 1 = yes; 2 = no.	+
Agricultural-related assistance	Dummy: 1= yes; 2 = no.	±
Marital status of household	Categorical: 1 = employed full time; 2 = employed part time; 3 = self-employed; 4 = not employed; 5 = studying full time.	±
Market distance	Continuous variable	±

**Table 3:** Demographic characteristics of Limpopo household.

Characteristics	n	%
Gender		
Male	944	46.2
Female	1099	53.8
Participant household distribution by study site		
Capricorn	382	18.7
Greater Sekhukhune	439	21.5
Mopani	434	21.2
Vhembe	425	20.8
Waterberg	363	17.8
Level of education		
Grade R/0 to grade 7/standard 5	464	27.3
Grade 8/standard 6 to grade 12/standard 10	716	42.2
NTC 1/N1 to NTC III/N3	30	1.7
NTC4/N4 to NTC 6/occupational certificate—NQF level 5	14	.8
Diploma with less than grade 12/std 10	6	.4
Higher/national/advanced certificate with grade 12/std 10	11	.6
Diploma with grade 12/std 10/occupational certificate—NQF 1	36	2.1
Higher diploma/occupational certificate (b-tech diploma)—N	18	1.2
Post-higher diploma (master’s diploma/master’s degree)—N	4	.2
Bachelor’s degree/occupational certificate—NQF level 7	25	1.5
Honours degree/postgraduate diploma/occupational certificate	7	.4
Doctoral degrees (doctoral diploma and Ph.D.)—NQF level 10	4	.2
No schooling	310	18.2
Employment		
Employed full-time	230	15.4
Employed part-time	105	7.0
Self-employed	86	5.8
Not employed	1055	70.7
Studying full-time	16	1.1
Marital status		
Legally married	608	38.0
Living together, like husband and wife	102	6.4
Divorced	28	1.8
Separated but still legally married	10	0.6
Widowed	334	20.9
Single but have lived together with someone as husband/wife	71	4.4
Single and have never been married/never lived together as husband/wife	466	27.9
Salaries and wages	372	23.2
Household income		
No income	22	1.5
Less R1500	328	21.7
1501–3000	588	38.9
3001–4500	281	18.6
4501–600	98	6.5
Greater than 6000	195	12.9
Access to land	666	36.0
Own land	592	88.9
Rent land	7	1.1
Tribal authority	23	3.5
State owned land	4	0.6
Other	40	6.0
Land used for the production of food and other agricultural products	551	82.5

**Table 4:** Fruits and vegetables grown and consumed by Limpopo households.

Fruits and Vegetables	Consumed (%)	Produced (%)
10.5 (n = 215)	Fruits	32.6 (n = 272)
21.7 (n = 428)	Vegetables	96.2 (n = 922)
Fruits and vegetables	96.8 (n = 704)	24.6 (n = 485)

**Table 5:** Determinant of HFIAS and consumption of fruits and vegetables (endogenous Poisson regression model).

Variable	Consumption of Fruits and Vegetables			HFIAS		
	Coef.	Std.	*p*-Value	Coef.	Std.	*p*-Value
Age	−0.002	0.010	0.803	0.010	0.005	0.068 **
Education of household head	−0.001	0.001	0.580	0.001	0.001	0.297
Employment status of household head	−0.009	0.051	0.852	−0.033	0.025	0.181
Gender	0.247	0.065	0.000 ***	−0.061	0.035	0.080 *
Household size	0.015	0.016	0.352	0.014	0.007	0.049 **
Did___work for salary/wages?	−0.139	0.109	0.200	−0.031	0.057	0.593
Is the household head a grant recipient?	−0.183	0.100	0.068 *	0.060	0.051	0.238
Did household receive any social grant for disability?	0.116	0.096	0.227	0.090	0.051	0.079 *
Access to land	0.058	0.114	0.608	−0.072	0.045	0.113
Agricultural-related assistance	−0.032	0.215	0.881	−0.085	0.118	0.471
Marital status of household head	0.024	0.018	0.179	0.013	0.011	0.228
Market distance	−0.003	0.006	0.570	−0.019	0.022	0.398
Involved in agricultural production	0.213	0.122	0.080 *			
Constant	−0.072	0.772	0.925	−3.273	0.472	0.000 ***
/athrho	0.760	0.111	0.000 ***			
/lnsigma	1.106	0.046	0.000 ***			
rho	0.641	0.000				
sigma	3.021	0.002				
Wald test of indep. eqns	47.24					
Prob > chi2		0.0000				
Log likelihood	−2194.984					
Wald chi2(12)	24.94	0.000				
Prob > chi2	0.0151 **					

Notes: dependent variable is HFIAS and consumption; ***, **, and * indicate significance at 1%, 5%, and 10% levels, respectively. Source: authors’ analysis.

**Table 6:** Treatment effects for the consumption of fruits and vegetables on household food security—Poisson regression with endogenous treatment effect.

Treatment Effects	Coefficient	Std.	*p*-Value
Average treatment effect (ATE)	5.103	1.137	0.000 ***
Average treatment on the treated (ATT)	6.371	1.462	0.000 ***

Notes: ***, indicate significance at 1% level. Source: authors’ analysis.

## Data Availability

Restrictions apply to the availability of these data. Data were obtained and is available from South African Vulnerability Assessment committee (SAVAC) secretarial.
